# Effects of early lactation body condition loss in dairy cows on serum lipid profiles and on oocyte and cumulus cell transcriptomes

**DOI:** 10.3168/jds.2022-21919

**Published:** 2022-08-06

**Authors:** Meghan L. Ruebel, Lilian Rigatto Martins, Peter Z. Schall, J. Richard Pursley, Keith E. Latham

**Affiliations:** 1 Department of Animal Science, Michigan State University, East Lansing 48824; 2 Reproductive and Developmental Sciences Program, Michigan State University, East Lansing 48824; 3 Comparative Medicine and Integrative Biology Program, Michigan State University, East Lansing 48824

**Keywords:** body condition loss, oocyte transcriptome, lipid metabolism, mitochondrial activity

## Abstract

The objective of this study was to determine the effect of early lactation body condition (BC) loss in multiparous dairy cows on serum lipids and the effect of these changes on oocyte and cumulus cell transcriptomes. Body condition loss in dairy cattle after parturition is associated with reduced fertility and increased pregnancy loss. The complex interplay between BC, nutrition, dry matter intake, milk production, and time of calving has presented a barrier to understanding mechanisms leading to reduced fertility. We identified cows that lost BC (L group; n = 10) or maintained or gained BC (M/G group; n = 8) during the first 27 to 33 d in milk and investigated changes in serum fatty acids and oocyte and cumulus cell transcriptomes at 75 to 81 d in milk. The L group had increased serum levels of nonesterified fatty acids and mead acid, and reduced serum levels of petroselaidic acid and behenic acid. Transcriptome analyses revealed 38 differentially expressed genes (DEG) in oocytes and 71 DEG in cumulus cells of L (n = 3) compared with M/G group (n = 3). Network analysis connected serum fatty acid changes to downstream effects including reduced inflammatory response and mitochondrial membrane depolarization, increased production of reactive oxygen species, and functions related to fatty acid metabolism and cytoplasmic organization in oocytes. These effects were associated with predicted effects on signaling in oocytes through calcium, insulin, O-GlcNAcase (OGA), fibroblast growth factor receptor 4 (FGF4R), peroxisome proliferator activated receptor gamma coactivator 1 α (PPARGC1A), and phospholipase D2 (PLD2) pathways, with a connection to the cumulus cell via calcium signaling. These results connect BC loss following parturition to changes in serum lipid levels, and changes potentially affecting oocyte quality; thus, these results provide new insight into mechanism of reduced fertility.

## INTRODUCTION

Fertility decreases following the first parturition in dairy cattle (Sartori et al., 2002). Although several problems may contribute to this reduction in fertility, oocyte and embryo quality are thought to be major factors (Sartori et al., 2010). In dairy cows, small decreases in pregnancy rate and increased pregnancy losses result in reduced average daily milk production of the herd, and increased culling and replacement costs (Giordano et al., 2011). The resulting loss in production represents a major financial burden on industry practitioners, creating great interest in identifying mechanisms to prevent such decreases in pregnancy rate.

Substantial body condition (**BC**) loss and negative energy balance (**NEB**) in dairy cows during early lactation are associated with reduced fertility, pregnancy loss, and periparturient health problems (Middleton et al., 2019). Elevated serum nonesterified fatty acid (**NEFA**) levels and reduced serum glucose levels associated with NEB are correlated with reduced pregnancy at first insemination (Garverick et al., 2013), and affect serum progesterone and probability of insemination (Lüttgenau et al., 2016). Approaches to mitigating BC loss can include dietary supplementation of carbohydrate, protein, and fatty acids (Adamiak et al., 2006; Fouladi-Nashta et al., 2009; Leroy et al., 2014; Moussa et al., 2015) to enhance energy balance, but may adversely affect the reproductive system. Indeed, fatty acids play key roles in ovarian folliculogenesis, corpus luteum function, granulosa cell function, hormone production, ovulation, and pregnancy (Armstrong et al., 2001; Leroy et al., 2005, 2014; Richardson et al., 2013; Baddela et al., 2020; Sharma et al., 2020). In vitro fatty acid supplementation of oocyte maturation medium can benefit oocytes and embryos derived formed therefrom (Aardema et al., 2011; Paczkowski et al., 2014; Marei et al., 2017). Dietary fatty acids can modify membrane compositions and lipid profiles of oocytes and cumulus cells and enhance oocyte quality, and rumen inert fat can increase embryo quality (Fouladi-Nashta et al., 2009; González-Serrano et al., 2015). Bovine oocyte quality is enhanced by oocyte fatty acid content (Warzych and Lipinska, 2020). However, dietary lipid supplementation benefits may be dose-dependent. Some studies report limited benefit of dietary lipid supplementation (Leroy et al., 2014). Other studies have reported that the ovary, and cumulus cells in particular, may buffer oocyte exposure to elevated serum lipid levels (Fouladi-Nashta et al., 2009; Aardema et al., 2019), even though follicular lipid concentrations vary with dietary lipid supplementation (Adamiak et al., 2005; Leroy et al., 2014). Excess serum lipid concentrations can be toxic to oocytes (Paczkowski et al., 2014) and negatively affect certain characteristics such as cryotolerance (Shehab-El-Deen et al., 2009). Dietary lipids can also affect serum insulin, leading to compromised oocyte quality (Adamiak et al., 2005; Garnsworthy et al., 2008) and can adversely affect granulosa and cumulus cells (Baddela et al., 2020; Sharma et al., 2020). There is considerable variation in reported effects, or lack thereof, of dietary lipids on embryo quality (Fouladi-Nashta et al., 2009). Such variability in outcomes across studies with dietary lipid supplementation is likely due to differences in the form of supplement (e.g., saturated versus unsaturated fatty acids, and rumen inertness), metabolism of the supplement provided (D’Occhio et al., 2019), effects of lipid ratios on lipid uptake in the ovary, the underlying BC, energy balance of the individual cow, the window of time when the supplement is provided, and differences in effects during pre- and postcalving periods (Leroy et al., 2014).

The apparent interaction of dietary lipids with BC (Adamiak et al., 2005) suggests that mobilization of endogenous lipid stores likely plays a key role on determining outcomes. In particular, NEFA mobilized from lipid stores during lactation (Wang et al., 2019) could be problematic for oocyte quality. Exposure of oocytes to excess elevated levels of NEFA or other lipids can result in adverse metabolic programming that persists in embryos after fertilization (Van Hoeck et al., 2011; Van Hoeck et al., 2013; Valckx et al., 2014; Desmet et al., 2020). Elevated serum NEFA can alter granulosa cell and cumulus cell phenotypes and thereby affect the oocyte, alter oocyte mitochondrial function, induce endoplasmic reticulum stress, increase production of damaging reactive oxygen species (**ROS**), and induce oocyte apoptosis, and thus are considered major culprits in reduced dairy cow fertility (Moussa et al., 2015; Baddela et al., 2020). Excess NEFA mobilized from endogenous stores could contribute to reduced pregnancy rates per AI via adverse effects on oocyte quality and embryo metabolic programming (Leroy et al., 2014).

We hypothesized that BC loss in multiparous dairy cows leads to elevated serum lipids and altered oocyte quality. To gain insight into the mechanisms responsible for reduced fertility associated with BC loss in dairy cattle and how this might be prevented, we identified cows displaying loss of BC (L group) and cows that maintained or gained BC (M/G group) during the first 27 to 33 DIM with no alterations to diet. Our objective was to determine changes in serum lipids and transcriptomes of oocytes and cumulus cells from single growing pre-ovulatory follicles (not superovulated) in lactating multiparous dairy cows treated with the fertility program Double Ovsynch.

## MATERIALS AND METHODS

### Animals and Handling

This study used randomly selected multiparous lactating dairy cows from February to August 2019, at a commercial dairy farm (Nobis Dairy Farm, St. Johns, MI). Cows were housed in freestall barns, fed a mixed ration once daily, and had free access to water and feed. The mixed ration consisted of wheat, corn, corn-soybean meal-based concentrates alfalfa silage, and supplements that were formulated to meet nutrient recommendations for lactating dairy cows (NRC Subcommittee on Dairy Cattle Nutrition, 2001). This included the feed supplement Energy Booster HP (Milk Specialties Global; 60–70% palmitic acid, 10–15% stearic acid, 5–10% oleic acid, and 2–4% myristic acid). All animal handling and procedures performed were approved by the Michigan State University Institutional Animal Care and Use Committee.

**Figure 1.**
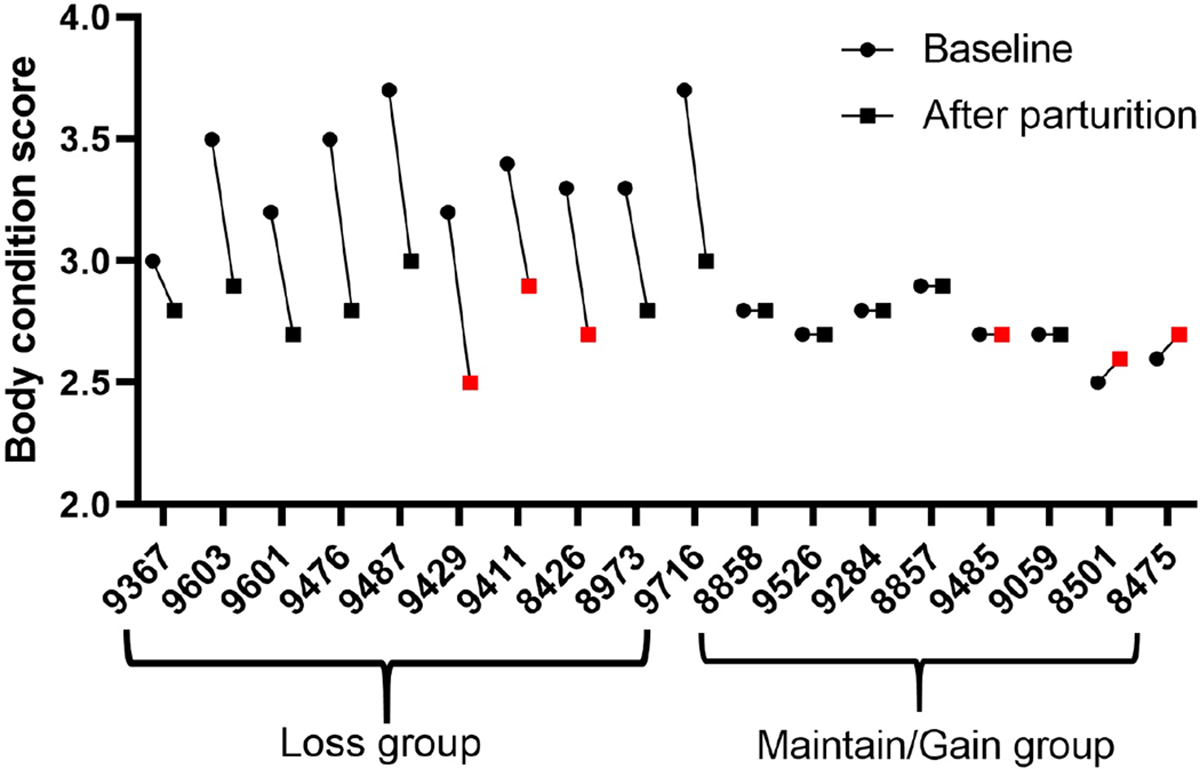
Changes in BCS for multiparous dairy cows that either lost (n = 10) or maintained or gained (n = 8) body condition during the first 27 to 33 DIM. Body condition scores were evaluated weekly according to a 5-point scale with 0.1-point increments. The measurement in the final week of gestation was set as baseline. Body condition scores were again determined at 27 to 33 d after calving to determine change within scores. Circles denote baseline BCS, and squares denote postparturition BCS. Red symbols denote the animals employed for RNA sequencing. The numbers on the x-axis are animal numbers.

Cows were divided into 2 groups based on BC change following parturition. Body condition of all multiparous cows <2 wk before expected time of parturition was evaluated according to a 5-point scale with 0.1-point increments and conducted weekly (Middleton et al., 2019). Body condition scores were determined within 1 wk of calving (baseline) and again at 27 to 33 DIM, and were used to determine change in BC during the first month of lactation. Cows that had a BCS loss following parturition to 27 to 33 DIM were put into the loss (**L**) group. All cows in this group had a ≥0.5-point BC loss with the exception of one cow with a 0.3-point loss during this period. Cows that maintained or increased their BC (≥0.1 point) from parturition to 27 to 33 DIM were put in the maintained/gained (**M/G**) group (Figure 1). Cows were synchronized using the Double Ovsynch program (Souza et al., 2008) beginning 48 to 54 DIM. Cows that had ovulation to each GnRH and CL regression following each PGF_2α_ during the Double Ovsynch program before final GnRH were used in this study. Thus, 10 cows remained in the L group, and 8 cows remained in the M/G group for data collection and analyses. The ovum pickup (**OPU**) procedure yielded 1 cumulus oocyte complex (**COC**) per cow, as expected from the single ovulatory follicle. We successfully recovered metaphase II (**MII**) oocytes (having first polar bodies) from 5 L group and 5 M/G group cows, and sufficient cumulus cell samples from 4 L group and 5 M/G group cows. Of the initial 10 MII oocytes and 9 cumulus samples included in the study, 4 oocyte and 3 cumulus samples were excluded due to low exonic read counts (<6 million), leaving 3 samples per condition for both cell types.

### Ovum Pickup

Ovum pickup was performed 20 h after the final GnRH of Double Ovsynch (75 to 81 DIM) to collect a single MII oocyte from cows with a single growing dominant follicle at the time of ovum pickup (Figure 1A). Cows were restrained in a chute and given caudal epidural anesthesia with lidocaine hydrochloride 2% (0.22 mg/kg; Lidocaine 2% Injection, VetOne) to allow handling of the ovaries through the rectum. The perineal area was cleaned using water and a preoperative surgical scrub brush (BD E-Z Scrub Brush, BD). The pre-ovulatory follicle was visualized using a real-time B-mode ultrasound scanner (Aloka SSD-500, Aloka) equipped with a 7.5-MHz microconvex transducer housed in a plastic vaginal probe. Follicular fluid and COC were aspirated into 50-mL conical tubes that contained approximately 3 mL of aspiration medium (HECM-HEPES supplemented with 0.3% BSA) using an 18-gauge × 75-mm disposable follicular aspiration needle (Partnar Animal Health). The needle was connected to a 1.1-mm inner diameter × 120-cm length Brazilian-style IVF tubing (Partnar Animal Health) inserted into a stainless-steel needle guide. Follicular aspiration was conducted using an electric suction pump (K-MAR-5200, Cook Medical) at a variable negative pressure of 280 ± 1 mmHg and adjusted to reach a flow rate of 30 to 35 mL of the aspiration medium per minute. The flow rate of aspiration medium through the system was measured for 1 min before each OPU. All OPU sessions were performed by the same operator.

### Serum Collection

Before epidural anesthesia for OPU (76–82 DIM), blood was collected by coccygeal venipuncture, allowed to clot for 30 min after collection, and transported to the laboratory. Blood samples were centrifuged at 2,000 × *g* for 20 min at 4°C. Samples were then aliquoted and stored at −80°C until analyses. Two milliliters of serum were submitted to the Michigan State University-Veterinary Diagnostic Laboratory (Lansing, MI) for the measurement of NEFA, total cholesterol, and TAG (n = 10 in the L and n = 8 M/G group) following standard procedures within the clinical pathology laboratory. Power analyses revealed that at least 8 cows per treatment were necessary for these fatty acid analyses (α = 0.05 and β = 0.25).

### Serum Fatty Acid Measurements

One milliliter of whole serum from each sample was extracted using a 2:1 ratio (vol/vol) of chloroform/methanol according to Folch et al. (1957), following previously established methods (Nuernberg et al., 2007; Bainbridge et al., 2015). Nitrogen gas was used to dry down the chloroform layer that contains the lipids, and resuspended in 1 mL of n-hexane/methyl tert-butyl ether/acetic acid (100:3:0.3; vol/vol). A solid-phase extraction with aminopropyl cartridges (Fisher Scientific) was used to separate the lipid classes. Columns were preconditioned with 1.2 mL of acetone/water (7:1; vol/vol) and 4 mL of n-hexane; next, the lipid solution was added. Fourteen milliliters of n-hexane was then added at a flow rate of 0.3 mL/min to elute cholesterol esters. Triglycerides (**TAG**) were extracted with 8 mL of n-hexane/chloroform/ethyl acetate (100:5:5; vol/vol). Then the column was rinsed with 6 mL of chloroform/isopropanol (2:1; vol/vol) and the solution was discarded. Free fatty acids and phospholipids (**PL**) were eluted by adding 8 mL of chloroform/methanol/acetic acid (100:2:2; vol/vol) and methanol/chloroform/water (10:5:4; vol/vol) to the column, respectively. Except for PL, all fractions were dried with nitrogen gas, resuspended in chloroform, and then stored at −20°C until transesterification. Phospholipids were instead washed with sodium chloride, dried down with nitrogen gas, re-dissolved in chloroform, and stored at −20°C. Following the procedures described earlier, the TAG fraction was methylated with sodium methoxide. Free fatty acids, cholesterol esters, and PL fractions were methylated with 1 mL of sodium hydroxide at 100°C for 10 min. Then 1 mL of 10% (wt/wt) boron trifluoride-methanol solution (Sigma-Aldrich) was added to the fractions and incubated at 100°C for 10 min. Fractions were then cooled to room temperature, and 2 mL of n-hexane and saturated potassium bicarbonate were added to them. We repeated the extraction step twice with 2 mL of n-hexane, then transferred over anhydrous sodium sulfate. All samples were then dried under nitrogen gas one last time and resuspended in 0.1% FAME solution of n-hexane and stored at −20°C until GC analyses.

A GC-2010 gas chromatograph (Shimadzu) equipped with a split injector (1:100 split ratio) and a flame ionization detector using an SP-2560 fused-silica capillary column (100 m × 0.25 m × 0.2 μm) was used to analyze fatty acids. The total fatty acid composition, covering 100 fatty acids that range from C:4 to C:24, was determined by GC analysis of FAME. This also included branched-chain fatty acids, isomers of octadecenoic acid (18:1), and conjugated linoleic acids. Hydrogen at a flow rate of 1 mL/min, and 40 mL/min for the flame ionization detector, was used as the carrier gas. The other gases were purified air at 400 mL/min and nitrogen gas at 30 mL/min. Temperature of 250°C was kept for both the injector and detector. The oven settings used for this experiment were as follows: initial temperature of 45°C, held for 4 min, programmed at 13°C/min to 175°C, held for 27 min, and then programmed at 4°C/min to 215°C and held for 35 min. One microliter of FAME mixture was used as injection volume. Using GC solution software (version 2.30.00), both integration and quantitation were calculated based on the flame ionized detector response. Fatty acid methyl esters were identified by comparison of retention times with known FAME standards (NuCheck Prep 463, 674, CLA mixture; Supelco PUFA-3 mixture and linoleic and linolenic acid mixture). The short-chain FAME were corrected for mass discrepancy using the established correction factors (Wolff et al., 1995; Bainbridge et al., 2015). Serum fatty acids were calculated as a percent of total fatty acid species recovered from serum in grams per 100 g. All values were calculated with an average response factor of 130 from external standards.

### Statistical Analyses for Serum Analyses

Serum lipids and fatty acid species measurements were expressed as means ± standard error of the mean, and comparisons between L and M/G cows, performed using Student’s *t*-test for parametric analyses, were used to assess differences between serum NEFA, total cholesterol, and TAG. Statistical significance was defined as *P* < 0.05. Statistical analyses were performed using GraphPad Prism 9.0. To assess the individual fatty acids that were changed between groups and take into account multiple testing, we used linear modeling. Measurements of fatty acids were imported into R (v 4.1.0) for statistical testing. Utilizing a binary coding of BC (0 = L, and 1 = M/G), a binomial generalized linear model was applied in a bidirectional stepwise method from a null to a full model equation, testing with a chi-squared metric. The resultant model equation identified 3 fatty acids (mead acid, behenic/docosonic acid, and petroselaidic acid) with an Akaike information criterion of 8.0 and residual deviance of 1.2 × 10^−9^. An ANOVA test comparing the null and full model equations resulted in a chi-squared value of 24.73 and a *P*-value of 1.76 × 10^−5^_._

### Oocyte and Cumulus Cell Isolation

The entire contents from each OPU collection tube (Corning Conical-Bottom 50-mL Tube) were transported in a portable incubator at 38.5°C to the laboratory for recovery of COC. Contents from the follicle were divided into 2 gridded 100-mm dishes, where 1 to 2 mL of fresh aspiration medium (Dulbecco’s PBS) supplemented with 100 μg/mL streptomycin sulfate, 100 units/mL penicillin G potassium, and 50 IU/mL heparin sodium was added. The conical tube was then rinsed with 200 mL of aspiration medium with heparin and poured into another gridded 100-mm dish. If a blood clot was present, it was immediately removed from the OPU collection contents and put into a separate dish filled with aspiration medium to minimize damage or loss of oocytes. Dishes were examined under a heated stereomicroscope to locate the COC. Using a Drummond Micropipette with glass tip, COC were collected and moved to a covered 35-mm dish filled with medium and kept at 38.5°C on a heated microscope stand. The COC were then transferred to hyaluronidase (Sigma-Aldrich; 300 μg/mL) to separate cumulus cells from oocytes. Oocytes were then washed through 3 ~ 150-μL drops of medium, and were transferred into a drop of acidified Tyrode’s buffer to remove zona pellucida, followed by a brief wash in medium. Finally, single MII oocytes were lysed in 20 μL of Pico Pure lysis buffer (Life Technologies) and heated treated at 42°C for 30 min before be storage at −80°C. Cumulus cells associated with each oocyte were washed 3 times in ~ 150-μL drops of Dulbecco’s PBS and lysed in 100 μL of PicoPure buffer and processed according to manufacturer’s instructions.

### RNA Sequencing and Transcriptome Analysis

For MII oocytes, total RNA was isolated from each oocyte using the PicoPure RNA isolation kit, following the manufacturer’s protocol, including a DNase digestion (RNase-Free DNase Set; Qiagen) to remove contaminating DNA. Oocyte libraries were constructed using Ovation SoLo RNA-Seq System (NuGen, now TECAN), including bead purification, end repair, adaptor ligation, and first round library amplification and purification steps. Then 20 to 30 ng of each library were used for the remainder of library preparation, which included use of AnyDeplete bovine primers for rRNA depletion, and a second round of library amplification and purification. Enzymatic shearing was applied, rendering all RNA-sequencing libraries between 300 and 350 bp in length. Barcoded libraries were pooled and sequenced on an Illumina HiSeq 4000 (Illumina) at the Michigan State University-Genomics Core (East Lansing). The NuGen Solo Kit control library was added to the sequencing lanes. The RNA sequencing of cumulus cell RNA was performed as above except that we used the NuGen (TECAN) Universal RNA-Seq with NuQuant kit. The RNA sequencing data are available at the Gene Expression Omnibus (GSE182151).

Raw sequencing data in FastQ format were initially queried for quality metrics using FastQC (version 0.11.9; https://www.bioinformatics.babraham.ac.uk/projects/fastqc). Trimming was conducted utilizing fastp (v 0.20.0) with the following settings: minimum Phred quality score threshold of 20, minimum read length of 20 base pairs, removal of low-complexity and polynucleotide reads, and hard trim first 3 base pairs on each read due to abnormal nucleotide distribution discovered via FastQC. The cow cDNA genome (ARS-UCD1.20, build 100) was downloaded from Ensembl and transcript abundances quantified with Kallisto (v 0.44.0) using standard settings.

Kallisto outputs were imported into R (v 4.0), and transcript abundances determined using Ensembl gene identifiers converted with biomartR (v 2.45.8). Cumulus and oocyte mRNA abundances were processed independently. Differential mRNA expression between groups was determined with DESeq2; specifically, genes with fragments per kilobase of transcript per million mapped reads (**FPKM**) greater than 1 in at least 1 sample were included. A positive log2 (fold-change) indicates a higher expression in L compared with M/G; moreover, we set the level of significance for genes at an adjusted *P*-value (false discovery rate) below 0.05. At a high sequencing depth, n = 3 oocytes per treatment is capable of revealing ~ 80% of affected mRNA between stages or conditions (Ching et al., 2014).

Ingenuity Pathway Analysis (**IPA**) was used to identify affected canonical pathways (CP), upstream regulators (**UR**), and biological functions (**BF**; subsets of IPA Disease and Functions outputs) using the differentially expressed gene (**DEG**) lists. Ingenuity Pathway Analysis uses a large manually curated database of >7 million observations combined with >30 other integrated databases to reveal predicted changes associated with specific CP, UR, and BF based on the level of significant over-representation (*P*-value; significance set at *P* < 0.05). Ingenuity Pathway Analysis also yields data on directionality (activated or inhibited) for some of these effects, expressed as a z-score (significance set at z > |1.96|). For UR, the IPA software leverages the known interactions (inhibits, activates, binds, phosphorylates, and so on) to predict the activity of regulators based on the direction of change of the submitted DEG. It should be noted that the magnitude of change of DEG does not factor into the calculations, solely the direction of change. Ingenuity Pathway Analysis entries with one DEG, UR that were drug or nonendogenous chemicals, and CP/BF related to cancer were excluded from the reported outputs. The analysis used the IPA database as of March 15, 2021.

An expanded network analysis was performed by integrating the IPA core analysis (CP/UR/BF) with information for increased versus decreased serum abundances of fatty acids in the L-group cows using the IPA Path Explorer. Based on the IPA Knowledge Base, predictions were derived for the activities of additional regulators that connect to identified DEG and affected regulators identified by UR analysis, providing connections to affected downstream functions along with additional predictions of overall activities. Regulatory nodes were identified and connected via the shortest path, and additional nodes were added as needed to connect affected fatty acids to identified oocyte and cumulus cell DEG. This method was also used to predict pathways by which affected fatty acids could be connected to differences in downstream functions. It is noted that, because this analysis examines connections between DEG and affected fatty acids in a more narrowly focused context, it can identify more upstream regulators with predicted increases or decreases in activities compared with the initial UR analysis alone.

### Supplemental Data

Supplemental data are available at Figshare (Supplemental Tables S1–S5; https://doi.org/10.6084/m9.figshare.15032037.v1; Latham et al., 2022). Sequencing data are available at the Gene Expression Omnibus accession number GSE182151.

## RESULTS

### Experimental Model

Multiparous lactating Holstein cows in the L group lost an average of 17% BC during the first 27 to 33 DIM, arriving at BCS similar to the persistent scores displayed in the M/G group (2.5% average increase; Figure 1). This indicates substantial mobilization of lipid stores in the L group.

### Comparison of Serum Lipid Profiles Between L and M/G Group Cows

**Figure 2.**
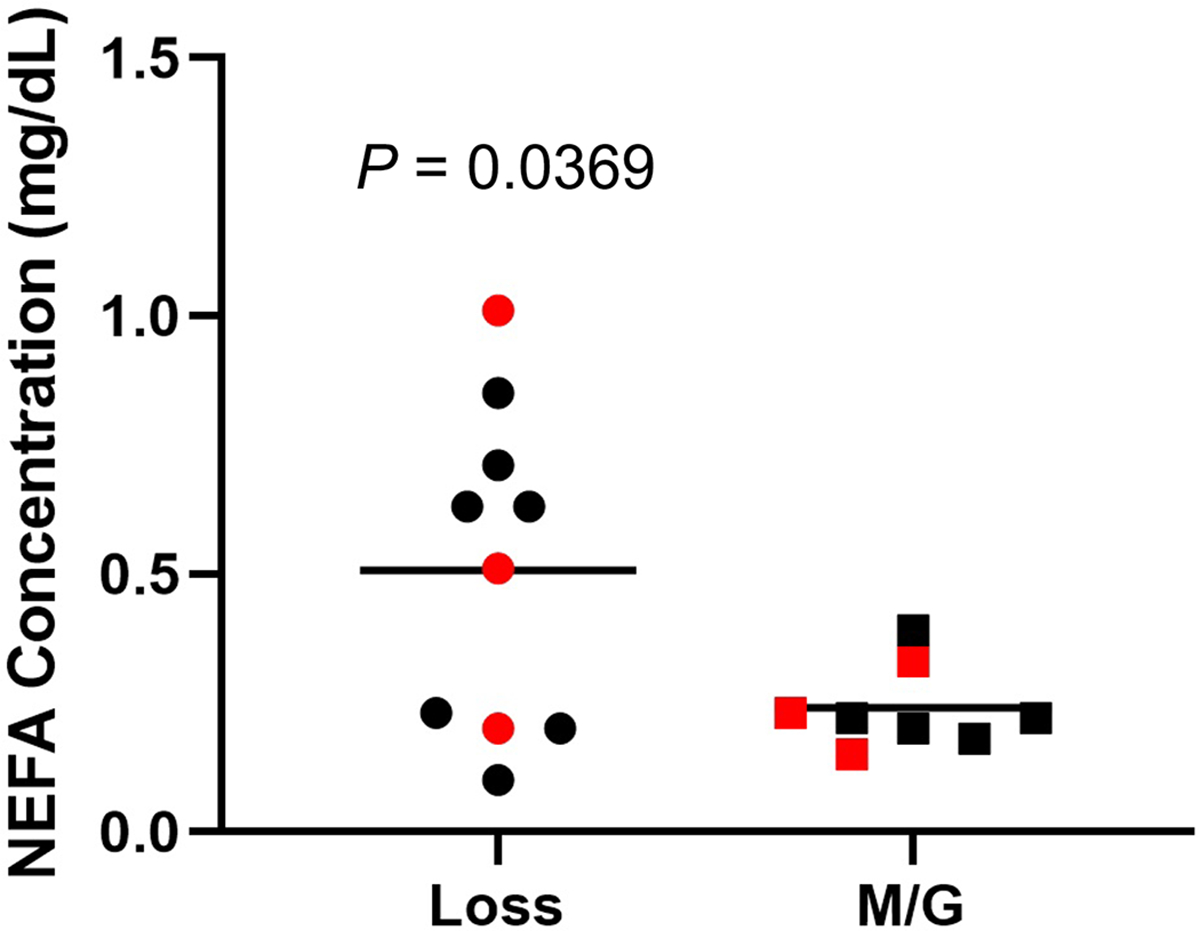
Serum levels of nonesterified fatty acids (NEFA) for lactating dairy cows that either lost (n = 10) or maintained or gained (M/G; n = 8) body condition were assessed from blood collected at 76 to 82 DIM during the first 27 to 33 DIM. A significant difference between groups was set at *P* < 0.05 using Student’s *t*-test. Circles denote loss group animals, and squares denote M/G group animals. Red symbols are those employed for RNA sequencing.

To determine whether BC loss in the L group was accompanied by elevated serum total NEFA levels, we analyzed serum NEFA in samples collected between 75 and 81 DIM. Average NEFA levels were elevated by more than 2-fold in the L compared with the M/G groups, although 4 of the L group cows had NEFA levels similar to those seen in the M/G group (*P* < 0.05; Figure 2). A further analysis of individual fatty acid species (Table 1; Supplemental Table S1) using linear modeling identified 3 individual fatty acids that were altered between L and M/G groups. Decreases in petroselaidic (18:1 6–8*t*, *P* = 0.0008) and behenic acid (22:0, *P* = 0.09) and an increase in mead acid (20:3 5*c*, 8*c*, 11*c*, *P* = 0.0001) were observed (Table 1).

### RNA Sequencing and IPA Analysis of Oocytes from L and M/G Groups

**Figure 3.**
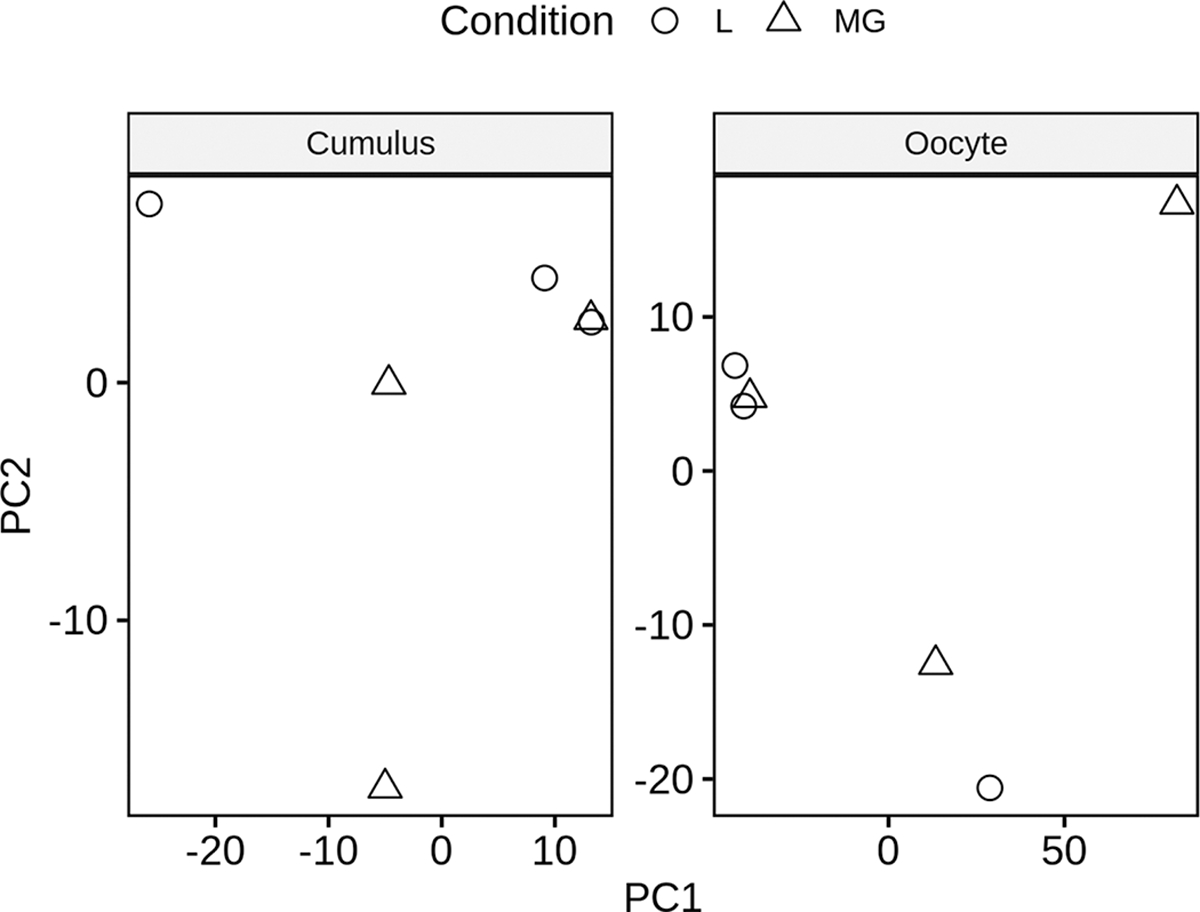
Principal component (PC) analysis of samples in RNA sequencing data set values after applying a variance stabilizing transformation within the DESeq2 package. Analysis indicates a very high degree of similarity between cows that lost (L) versus maintained or gained (M/G) body condition during the first 27 to 33 DIM.

**Figure 4.**
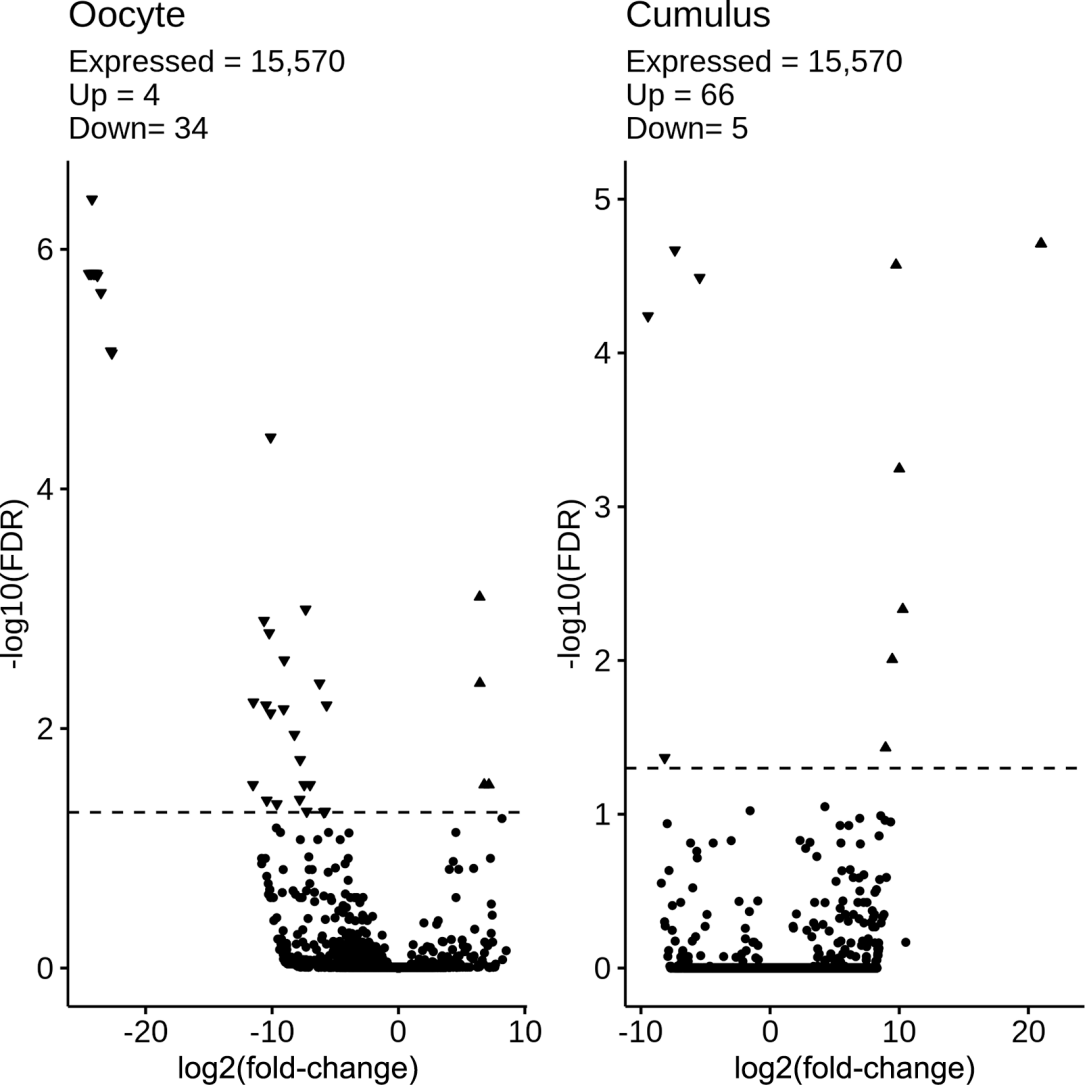
Volcano plots depicting comparisons of oocyte and cumulus cell transcriptomes from cows that lost (L; n = 3) versus maintained or gained (M/G; n = 3) body condition during the first 27 to 33 DIM. Figure contains panels for oocytes (left) and cumulus cells (right). The log_2_(fold-change) comparing L versus M/G is along the x-axis, and the −log_10_(FDR) along the y-axis, significance represented by horizontal dashed line at 1.3, equating to −log_10_(FDR = 0.05). Shape denotes direction of change and significance: circle = nonsignificant difference; uptriangle = upregulated in L; and down-triangle = downregulated in M/G. FDR = false discovery rate.

Suitable high-quality RNA sequencing results were obtained for libraries produced from a total of 3 single MII stage oocytes from L group (average of 57.2 million passed-filter reads and 26.5 million aligned exon reads per library), and the 3 single MII stage oocytes from M/G cows (average of 49.7 million passed-filter reads and 19.7 million aligned exon reads; Supplemental Table S2). The 3 L group cows averaged more than 2-fold higher mead acid, 3-fold lower behenic acid, and 43% lower petroselaidic acid, and 2 displayed highly elevated NEFA levels compared with the M/G cows (Figure 2). Overall, an average of 14,432 genes were captured in the detected oocyte mRNA populations (Supplemental Table S2). Principal component analysis (Figure 3) indicated a lack of clear separation between the 2 sample groups, indicating a high degree of similarity between the 2 groups. Additionally, Pearson correlation coefficients ranged from 0.53 to 0.97 for all pair-wise comparisons. Consistent with this, comparing the L versus M/G samples resulted in the identification of just 38 total DEG, 4 upregulated, and 34 downregulated in the L samples (Figure 4; Supplemental Table S3). Interestingly, many of the DEG displayed large, essentially qualitative differences in expression (i.e., not detected in one group).

**Figure 5.**
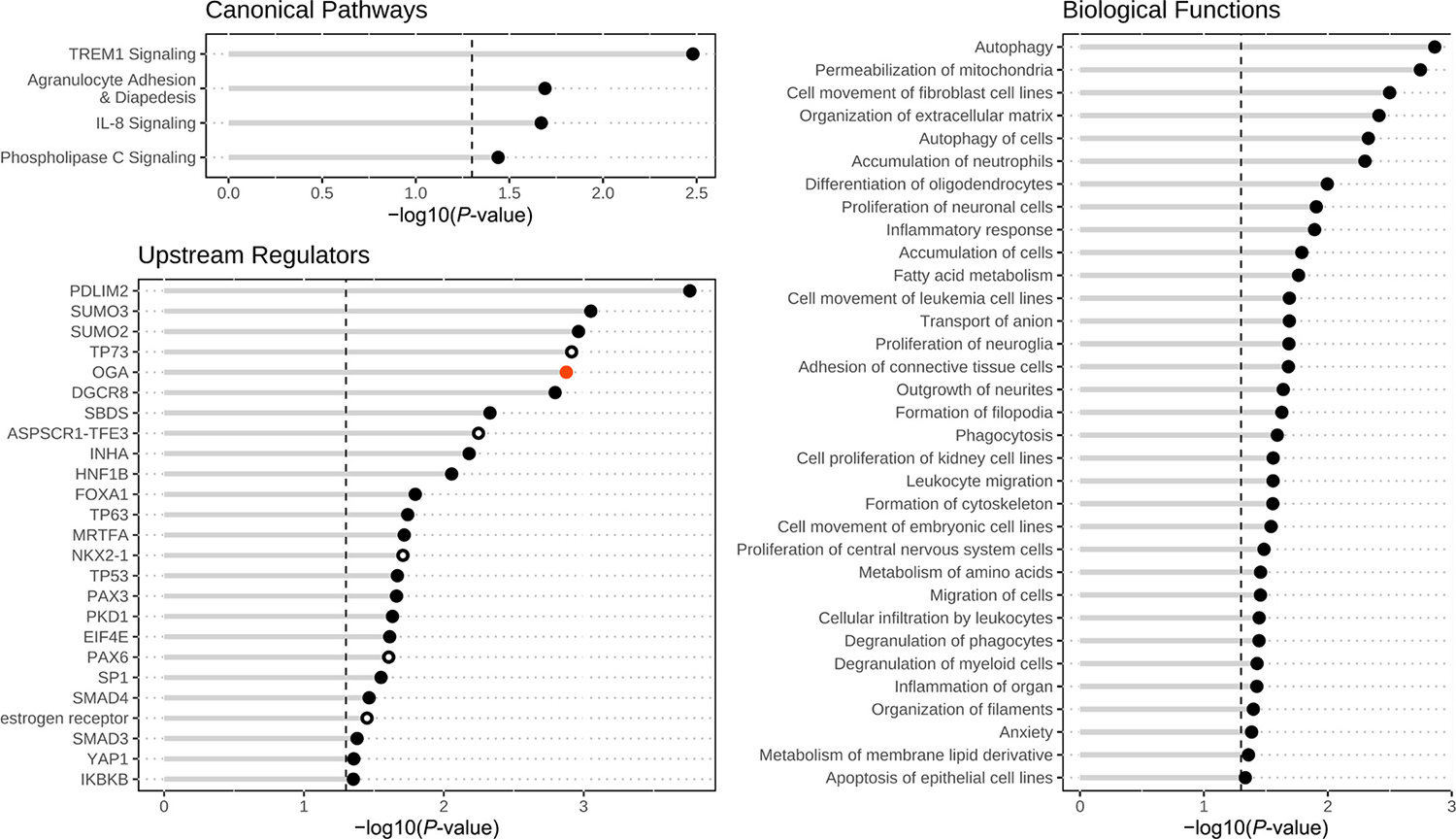
Effects of body condition loss on transcriptomes for lactating dairy cows that either lost or maintained or gained body condition during the first 27 to 33 DIM, as observed using Ingenuity Pathway Analysis (IPA). Canonical pathways, biological functions, and upstream regulators were identified by IPA as being significantly affected. Black closed circles denote IPA pathways, functions, or upstream regulators that are significantly affected. The red closed circle denotes an upstream regulator predicted to be activated. Open circles denote upstream regulators predicted to be affected but not expressed in the oocyte. Details of these effects are shown in Supplemental Table S3.

To identify pathways, regulators, and functions associated with these transcriptome changes, the oocyte DEG were submitted to IPA core analysis. Four significantly affected CP, each with 2 associated downregulated DEG, were identified (Figure 5; Supplemental Table S4). We identified 25 affected UR, with highest confidence values assigned to PDZ and LIM domain protein 2 (PDLIM2), small ubiquitin-like modifiers 2 and 3 (SUMO2, SUMO3), tumor protein 73 (TP73), and O-GlcNAcase (OGA), whereas tumor proteins 53 and 73 (TP53, TP73), OGA, PDLIM2, tumor protein 63 (TP63), and transcription factor SP1 (SP1) had the largest number of affected downstream target DEG. The protein OGA was associated with a significant positive z-score, indicating predicted activation of signaling (Supplemental Table S4; Figure 5). The BF analysis yielded 33 significant entries, with the most prominent being effects on autophagy, permeabilization of mitochondria, fatty acid metabolism, inflammatory response, glucose tolerance, and numerous entries related to cell proliferation, migration, and cytoskeleton (Figure 5; Supplemental Table S4). Biological functions related to autophagy, inflammation, cell migration, and fatty acid metabolism had the largest numbers of associated DEG (Supplemental Table S4).

**Figure 6.**
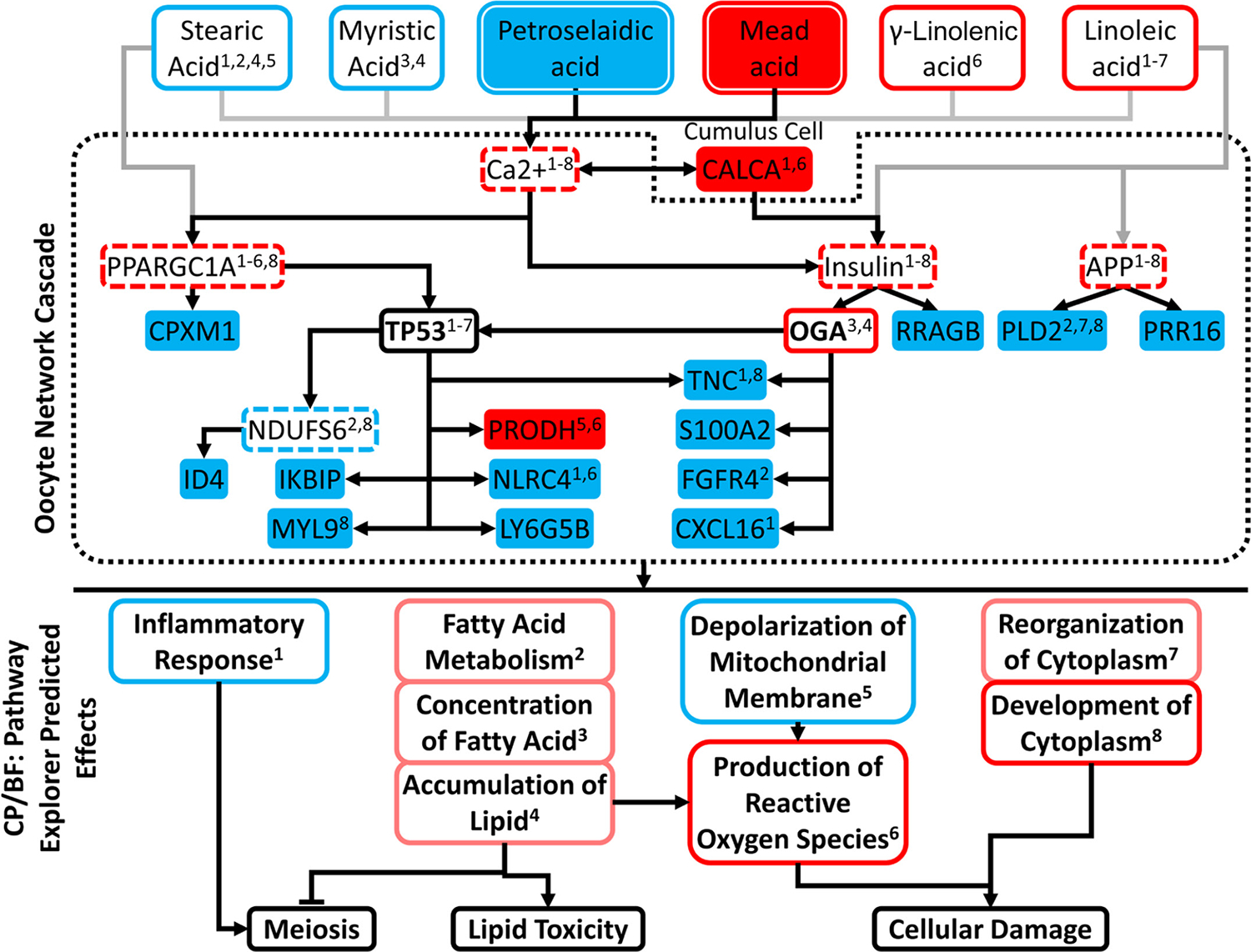
Expanded network analysis linking fatty acids with significantly altered serum levels, upstream regulators, downstream differently expressed gene (DEG) targets, and their enrichment within pathways and functions. Figure consists of 3 boxed tiers: (1) Fatty acids significantly different between the loss (L) and maintain or gained (M/G) groups or with predicted activation/inhibition; (2) upstream regulators downstream of fatty acids and the DEG targets by said regulators; and (3) enriched canonical pathways (CP) and biological functions (BF). Factor not bounded by central dotted box (CALCA) denotes a cumulus cell DEG connecting to Ca^2+^ and insulin. Color fill of nodes denotes measured significant difference between L and M/G: red = increased in L; blue = decreased in L. Exterior color denotes Ingenuity Pathway Analysis (IPA) predicted z-score: red = activated; blue = inhibited; pink = positive trend not meeting significance. Regulators with bold font (OGA and TP53) denote those upstream regulators (UR) that were identified from IPA core analysis as significantly affected. Regulators with dashed red lines are those identified by the IPA Path Explorer tool to connect fatty acids to other regulators and DEG, but not initially indicated by the IPA core analysis. Gray lines indicate possible effects emanating from lipids that did not reach statistical significance for difference in serum concentrations. CP/BF membership of fatty acids, UR, and DEG denoted by superscript, corresponding to matched CP/BF superscript.

We next performed an expanded network analysis by integrating data for specific affected lipids (Table 1) with IPA results and using the IPA Path Explorer tool to identify additional UR that connect these lipid changes to identify downstream DEG and affected pathways and functions (Figure 6). Six individual fatty acids, including 2 that were significantly altered in the L group, were associated with predicted downstream effects via signaling through 4 UR predicted to have increased activities using Path Explorer. These 4 regulators were not identified using the initial IPA UR analysis tool, which did not incorporate possible oocyte exposure to altered serum lipids. The 4 UR included calcium, which is predicted to affect 2 of the other regulators, peroxisome proliferator activated receptor gamma coactivator 1 α (PPARGC1A), and insulin. These 3 regulators were predicted to affect 1 overexpressed and 11 underexpressed DEG by signaling through TP53, which was predicted to affect 7 affected downstream CP/BF, and OGA. This last, in turn, was also predicted by UR analysis to be activated and had predicted direct or indirect effects on 6 affected CP/BF. A fourth predicted regulator, APP, was predicted to be activated, with signaling through phospholipase D2 (PLD2) and proline-rich 16 (PRR16) protein, and with effects on 3 affected CP/BF via reduced PLD2 expression.

This analysis yielded 8 affected CP/BF, 2 with significant predicted inhibition (inflammatory response, mitochondrial membrane depolarization), 2 with significant predicted activation (development of cytoplasm and production of ROS), and 4 marginally activated, 3 of which were related to fatty acid metabolism and 1 related to cytoplasmic organization.

### RNA Sequencing and IPA Analysis of Cumulus Cells from L and M/G Group Cows

We also compared transcriptomes of cumulus cells between L and M/G groups. Principal component analysis (Figure 3) revealed a lack of clear separation between the 2 sample groups, indicating a high degree of similarity between the 2 groups. Additionally, Pearson correlation coefficients ranged from 0.68 to 0.96 for all pair-wise comparisons. An average of 13,484 genes were captured among the samples analyzed (Table S2). A total of 71 DEG were identified for cumulus cells, of which 66 were higher in expression in the L group (Figure 4, Supplemental Table S5). However, most of these DEG were undetected in M/G group samples and expressed at relatively low levels (<5 FPKM) in the L group samples and were not considered further. Four DEG were expressed above 5 FPKM and were higher in M/G samples, and 20 were expressed above 5 FPKM and were higher in L group samples. Because only 5 of these 24 DEG were assigned gene symbols, a separate IPA core analysis to identify affected CP/UR/BF was not performed. However, IPA Path Explorer revealed that calcitonin/calcitonin related polypeptide α (CALCA) could affect oocytes through its effects on calcium and the pathways downstream of calcium, shown in Figure 6.

## DISCUSSION

The results presented here provide new insight into the mechanisms that contribute to reduced fertility in dairy cows that undergo substantial BC loss during lactation. The L group displayed initial BC scores of 3 to 3.75 on a 5-point scale on day of parturition. Similar cows in an earlier study displayed elevated serum leptin and NEFA levels (Wang et al., 2019). As such, the L group most closely resembled a condition of relative undernutrition or NEB during lactation after losing approximately 17% of BC during the first month postparturition. Additionally, the results reveal an elevation of serum NEFA and mead acid and a decrease in petroselaidic and behinic acid.

The increase in NEFA seen during BC loss and early lactation of dairy cows is due to an increase in lipolysis and decrease in fatty acid re-esterification (Chilliard et al., 1998, 2000), which leads to a change in fatty acid levels. In this study, we observed 3 individual fatty acids that were altered in our linear model analysis, including decreased serum levels of petroselaidic (18:1 6–8t, *P* = 0.0008) and behinic acid (22:0, *P* = 0.09) and increased serum mead acid (20:3 5c, 8c, 11c, *P* = 0.0001) in the L group compared with the M/G group. Mobilization of body fat leads to an increase in long-chain serum fatty acids, likely accounting for the increases of some serum fatty acid levels seen in this study (Andres Contreras et al., 2020). Mead acid, which was elevated in the L group, is associated with increased intrauterine growth restriction when elevated, but has not been shown to affect oocyte, cumulus cell, or embryo development (Powell et al., 1999; Chassen et al., 2018). Behenic acid is present in both serum and follicular fluid of dairy cows, but both decreased and increased serum levels of this fatty acid have been reported to be associated with reduced fertility (Bender et al., 2010; Moore et al., 2017). Petroselaidic acid is a *trans*-fatty acid isomer, for which lower levels can affect ovulation rates and pregnancy outcomes in humans (Kim et al., 2019; unpublished data by M. Li, Y. Tian, Y. Lv, Y. Xu, X. Bai, H. Zhang, Y. Wang, and X. Song, Tianjin Medical University General Hospital, Tianjin, China). Increases of *trans*-fatty acids in fluidity of membrane lipid composition could have adverse effects on oocyte quality (Çekici and Akdevelioglu, 2019). Overall, the observed changes in specific serum fatty acids could negatively affect ovulation rate, oocyte quality, pregnancy outcomes, and development of the offspring in L-group animals, although further studies would be needed to confirm this effect.

A recent study comparing dairy cattle at different times during lactation concluded that during early lactation, elevated serum fatty acids were associated with elevated oocyte TAG (Furukawa et al., 2021). This is consistent with the possibility that elevated serum lipids can affect the oocyte. The effects of serum fatty acids seen here could alter the intracellular ratios of fatty acids and oocyte lipid content, resulting ultimately in the downstream effects reflected in the transcriptome. It has been suggested that conversion of fatty acids to triacylglycerol, a less harmful lipid form than free fatty acids, could be protective to the oocyte (Furukawa et al., 2021). However, the apparent changes in the oocyte transcriptome suggest that, if conversion of fatty acids to triacylglycerol is occurring in L-group oocytes, it may be insufficient to fully mitigate harmful effects of changes in serum and oocyte lipid profiles.

**Figure 7.**
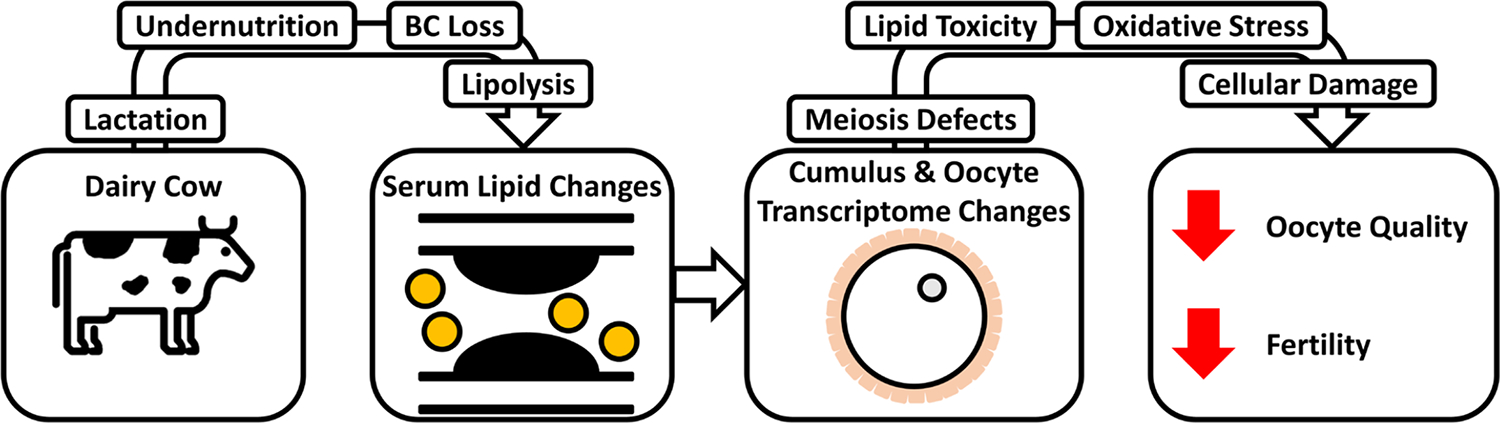
Working model of how negative energy balance in lactating dairy cows ultimately affects oocyte quality and fertility. Lactation in animals in the loss group leads to a condition of negative energy balance and body condition (BC) loss with lipolysis. This changes serum lipid profiles, which in turn lead to transcriptome changes associated with possible meiosis defects, lipid toxicity, oxidative stress, and cellular damage, reducing oocyte quality and fertility.

Because ovarian follicular lipids change in concert with serum lipid levels, albeit with some buffering (Leroy et al., 2005), such changes in serum lipids could affect oocytes. Oocyte maturation and developmental competence are affected by individual fatty acids in multiple animal species (Leroy et al., 2005; Dunning et al., 2014; Nagano, 2019; Warzych and Lipinska, 2020). Effects include inhibition of cumulus expansion, delayed maturation, apoptosis, changes in energy metabolism and oxidative stress related gene expression, ER stress, and mitochondria effects in oocyte or cumulus cells, or both (Leroy et al., 2005; Van Hoeck et al., 2011, 2013; Wu et al., 2012; Dunning et al., 2014). Additionally, increases in the oocyte content for certain longer-chain fatty acids are associated with oocyte degeneration (Chen et al., 2020). We propose a possible mechanistic model of how excessive BC loss reduces dairy cow fertility based on serum lipid analyses and limited RNA sequencing analyses of oocytes and cumulus cells in cows that lose versus maintain or gain BC during the first 27 to 33 DIM (Figure 7). The model connects changes in serum lipids to downstream negative effects on oocyte quality and fertility arising through 4 molecular and cellular mechanisms: increased ROS production, aberrant cellular cytoskeletal organization, lipid toxicity, and disruptions in meiosis regulation (Figures 6 and 7).

Previous studies have demonstrated that changes in oxidative phosphorylation, production of ROS, lipid toxicity, and mitochondrial function can alter oocyte competence, quality, or both, and serum lipids and fatty acids may play a role in these processes (Penzo et al., 2002; Zorova et al., 2018; Ruebel et al., 2018, 2021; Warzych and Lipinska, 2020). Body condition loss in the L group is proposed to lead to enhanced lipolysis, altering serum fatty acid profiles as reported (Andres Contreras and Sordillo, 2011; D’Occhio et al., 2019; Gross and Bruckmaier, 2019). Fatty acids, particularly longer and with greater degrees of unsaturation, can reduce mitochondrial membrane depolarization and cell viability and increase ROS production in bovine oocytes (Penzo et al., 2002; Marei et al., 2012, 2019). Our analysis indicated that reduced mitochondrial membrane depolarization, accompanied by increased ROS production and fatty acids, could lead to changes in these biological processes. Because depolarization is one mechanism to reduce ROS production (Zorova et al., 2018), we propose that the combination of serum lipid changes inhibits this function and precludes homeostatic regulation of mitochondrial membrane potential. This could increase ROS production and affect mitochondrial viability, either of which could be detrimental to L-group oocytes (Figure 7).

The requirement for oocyte lipid content varies with species, with bovine oocytes benefitting from higher fatty acid availability (Warzych and Lipinska, 2020). The predicted increase in fatty acid synthesis in oocytes of L-group cows could thus be viewed as potentially beneficial to the oocyte. Although increased NEFA availability can promote fatty acid metabolism and β-oxidation, which could be beneficial to oocytes, excessive NEFA can inhibit fatty acid translocation into mitochondria, which could be detrimental (Li et al., 2013). Therefore, the predicted increase in lipid accumulation (Figure 6) could indicate another contributor to reduced oocyte quality (Figure 7).

Several effects on meiosis are predicted in our analysis. Lipopolysaccharide-mediated activation of the inflammatory response in oocytes is detrimental to oocyte cytoplasmic maturation (Zhao et al., 2019). The predicted inhibition of the inflammatory response (Figures 6 and 7) observed here could reflect a protective mechanism activated under the conditions of altered serum lipids, or a more generalized systemic inhibition of inflammatory response in the L-group cows. Alternatively, the predicted inhibition of inflammatory response could reflect modulations of diverse signaling pathways such as toll receptor signaling, NFkB signaling, and nitric oxide signaling, all of which have key roles in the regulation of oocyte functions (Liu et al., 2008; Nath and Maitra, 2019; Zhao et al., 2019; Gao et al., 2019).

Our analysis also implicates specific regulatory molecules in mediating the predicted negative effects on the oocyte phenotype. One of the most prominent and potent regulators predicted to be affected with BC loss in oocytes is OGA. The protein OGA emerged here with a strong predicted activation through IPA UR analysis as well as Path Explorer analysis, and as a key upstream regulator predicted to directly or indirectly affect 6 of the 8 CP/BF outlined in Figure 6. O-linked N-acetyl glucosamine (O-GlcNAc) protein modifications play key roles in many diverse cellular processes, but in particular functions in nutrient and stress sensing to regulate signaling, transcription, mitochondrial activity, and cytoskeletal functions (Hart, 2019). An imbalance between OGT [O-linked N-acetylglucosamine (GlcNAc) transferase] and OGA can lead to serious physiological effects and diseases (Hart, 2019). In other contexts, increased OGA activity can reverse negative effects of excess O-GlycNAc deposition (Hart, 2019), such as the negative effects predicted here on mitochondrial membrane polarization and mitochondrial function. O-GlcNAcylation may also modulate the function of the oocyte proteins. Specifically, it has been shown to have an important role in meiotic maturation and fertilization (Shibutani et al., 2015; Slawson and Duncan, 2015; Zhou et al., 2019).

A second prominent regulator was calcium, predicted to operate through insulin and PPARGC1A to regulate many of the predicted effects on downstream CP and BF (Figure 6). Insulin can affect most of the CP and BF associated in Figure 6. PPARGC1A is a master regulator of mitochondrial biogenesis; its predicted activation here could provide the oocyte with another means of overcoming negative effects of BC loss on mitochondrial function. However, the effects observed also lead to excess production of ROS, which would be detrimental in oocytes. Indeed, a key part of oocyte maturation across mammalian species appears to be the degradation of mRNA associated with oxidative phosphorylation (Ruebel et al., 2018, 2021; Schall and Latham, 2021).

The reduced expression of PLD2 could also play an important role in compromised oocyte health. Reduced expression of PLD2 could negatively affect essential cytoskeletal functions and cellular organization, cell signaling related to metabolic functions, immune functions, and many other cellular functions that depend on correct lipid signaling (Rudge and Wakelam, 2009; Gomez-Cambronero, 2010, 2014).

The potential effect of fatty acids on cumulus cell-oocyte calcium signaling via CALCA may be a key part of the mechanism linking changes in serum fatty acids to effects on oocyte viability. Although the follicle buffers serum lipid exposures of oocytes (Fouladi-Nashta et al., 2009), serum lipid effects on cumulus cells (Marei et al., 2019), including calcium signaling, would provide an important means by which the oocyte could be affected.

One striking aspect of the results of our transcriptome analysis is the predicted effects on key regulators that serve sweeping, fundamental roles in diverse functions across many cell types, such as OGA, PLD2, and PPARGC1A, insulin, and calcium. The high serum leptin levels previously reported, combined with elevated serum NEFA and fatty acids and changes in the oocyte transcriptome reported here, indicate that lactating dairy cows likely face unique challenges affecting overall fertility. Oocyte quality may be severely compromised, with effects on metabolic and homeostatic pathways. Additional detrimental effects on immune system function leading to further reproductive effects of infections could also work additively with impaired oocyte quality to reduce overall reproductive performance. Physiological effects in these cows via changes in insulin signaling and changes in O-GlcNAc deposition within cells, particularly in brain and cardiac tissues, could also affect overall reproductive success.

Overall, our results indicate significant changes in serum lipid profiles in the L group and significant associated oocyte and cumulus cell transcriptome changes predicted to affect the oocyte phenotype (Figure 7). Such alterations in serum lipid profile are associated here with potential detrimental effects on oocyte physiology. It is interesting to note that the L-group cows display such signs of undernutrition even though all cows in this study were provided a fatty acid supplement (see Materials and Methods), indicating that such dietary supplementation did not prevent excessive BC loss and associated effects on the oocyte. These observations indicate that complete understanding of the mechanisms causing excessive BC loss and the mechanisms by which BC loss affects dairy cow reproduction and fertility will require study of multiple physiological functions. Furthermore, the findings here highlight the importance of achieving a better understanding of how complex physiological changes in serum lipid profiles might interact with dietary fat supplements and affect oocyte quality, as previously suggested (Leroy et al., 2014). The effects shown here for oocytes comprise an important piece of that much larger puzzle.

## Figures and Tables

**Table 1.** Effects of body condition loss between calving and 27 to 33 DIM on serum lipids and fatty acid concentrations in lactating dairy cow between 75 and 81 DIM from loss (L) and maintain or gain (M/G) groups

Fatty acid	Name	L^1,2^	M/G^1,2^	*P*-value^3^	Ratio (L:M/G)^4^

C18:1 6–8*t*	Petroselaidic acid	0.097	0.125	0.0008*	0.78
C20:3 5*c*,8*c*,11*c* (n-9)	Mead acid	0.066	0.036	0.0001*	1.83
C22:0	Behenic, docosanoic acid	0.02	0.032	0.09	0.63
Sum of fatty acids^5^					
Σ Unknown		0.516	0.675	0.0332*	0.76
Σ SFA		36.45	30.92	0.0343*	1.18
Σ MUFA (n-7 and n-9)		13.94	14.1	0.8905	0.99
Σ PUFA (n-3 and n-6)		47.21	35.68	0.0373*	1.32
Σ n-3		1.954	1.321	0.159	1.48
Σ n-6		45.19	34.33	0.0346*	1.32
Serum markers^5^					
Total cholesterol^6^		229.6	225.4	0.8921	1.02
Triglyceride^6^		10.9	9.25	0.1215	1.18

1 Fatty acid composition (grams per 100 g of fatty acids) of serum from dairy cows within the L (n = 10) and M/G (n = 8) groups.

2 Values are the mean (average).

3 Signifiance was determined using a binomial generalized linear model (binary coding of body condition: 0 = L, and 1 = M/G) in a bidirectional stepwise method from a null to a full model equation, testing with a chi-squared metric.

4 Ratio of means between L and M/G group.

5 A significant difference between groups was set at *P* < 0.05 using Student’s *t*-test (parametric).

6 Total cholesterol and triglyceride measured in mg/dL.

* Denotes *P* < 0.05.
